# Vitamin D Deficiency Induces Elevated Oxidative and Biomechanical Damage in Coronary Arterioles in Male Rats

**DOI:** 10.3390/antiox9100997

**Published:** 2020-10-15

**Authors:** Réka Eszter Sziva, Zoltán Fontányi, Éva Pál, Leila Hadjadj, Anna Monori-Kiss, Eszter Mária Horváth, Rita Benkő, Attila Magyar, Andrea Heinzlmann, Zoltán Benyó, György L. Nádasy, Szabolcs Várbíró

**Affiliations:** 1 Department of Obstetrics and Gynaecology, Semmelweis University, Üllői Street 78/a, 1082 Budapest, Hungary; fontanyi.zoltan@med.semmelweis-univ.hu (Z.F.); varbiro.szabolcs@med.semmelweis-univ.hu (S.V.); 2 Institute of Translational Medicine, Semmelweis University, Tűzoltó Street 37-47, 1094 Budapest, Hungary; pal.eva@med.semmelweis-univ.hu (É.P.); leila.hadjadj@gmail.com (L.H.); anna.monorikiss@gmail.com (A.M.-K.); benyo.zoltan@med.semmelweis-univ.hu (Z.B.); 3 Department of Physiology, Semmelweis University, Tűzoltó Street 37-47, 1094 Budapest, Hungary; horvath.eszter@med.semmelweis-univ.hu (E.M.H.); benko.rita@med.semmelweis-univ.hu (R.B.); nadasy.gyorgy@med.semmelweis-univ.hu (G.L.N.); 4 Department of Anatomy, Histology and Embriology, Semmelweis University, Tűzoltó Street 58, 1094 Budapest, Hungary; magyar.attila@med.semmelweis-univ.hu; 5 Department of Anatomy and Histology, University of Veterinary Medicine, István Street 2, 1078 Budapest, Hungary; hmannandrea@gmail.com

**Keywords:** vitamin D, rat model, cardiovascular disease, oxidative damage, vitamin D deficiency, male

## Abstract

Background: Several reports prove interconnection between vitamin D (VD) deficiency and increased cardiovascular risk. Our aim was to investigate the effects of VD status on biomechanical and oxidative–nitrative (O–N) stress parameters of coronary arterioles in rats. Methods: 4-week-old male Wistar rats were divided into a control group (11 animals) with optimal VD supply (300 IU/kgbw/day) and a VD-deficient group (11 animals, <5 IU/kg/day). After 8 weeks, coronary arteriole segments were prepared. Geometrical, elastic, and biomechanical characteristics were measured by in vitro arteriography. O–N stress markers were investigated by immunohistochemistry. Results: Inner radius decreased; wall thickness and wall-thickness/lumen diameter ratio increased; tangential wall stress and elastic modulus were reduced in VD-deficient group. No difference could be found in wall-cross-sectional area, intima-media area %. While the elastic elements of the vessel wall decreased, the α-smooth muscle actin (α-SMA) immunostaining intensity showed no changes. Significant elevation was found in the lipid peroxidation marker of 4-hidroxy-2-nonenal (HNE), while other O–N stress markers staining intensity (poly(ADP)ribose, 3-nitrotyrosine) did not change. Conclusions: Inward eutrophic remodeling has developed. The potential background of these impairments may involve the initial change in oxidative damage markers (HNE). These mechanisms can contribute to the increased incidence of the cardiovascular diseases in VD deficiency.

## 1. Introduction

Insufficient vitamin D supply and vitamin D deficiency are important public health problems worldwide with a continuously increasing prevalence in the general population [1]. Vitamin D is a fat-soluble and evolutionally well-conserved in vivo steroid product of almost all creatures on Earth [2]. Vitamin D has skeletal and extraskeletal effects. Another categorization of the effects of vitamin D are classical and non-classical actions. Three non-classic actions can be distinguished: (I) regulation of hormone secretion, (II) immune system modulation, and (III) regulation of cell proliferation and differentiation [3]. While the skeletal or classical targets are well-known, bone growth, calcium-phosphate homeostasis, the various exact extraskeletal actions on different organs and organ-systems are less known. The extraskeletal effects include the impact of vitamin D on skin, muscles, and the immune system. Alteration of vitamin D metabolism was shown to play role in the development of various pathological states, such as malignant diseases, metabolic disturbances (e.g., diabetes, obesity, metabolic syndrome), neurologic disorders, reproduction, liver, lung, and kidney diseases, mortality, cardiovascular system, and hypertension [4,5]. One important extraskeletal effect of vitamin D is its beneficial influence on the cardiovascular system [6]. 

Vitamin D makes impact through vitamin D receptor (VDR), which is the member of the nuclear receptor sub-family (NR1H), together with the liver X receptor α and β (LXRα/β), farnesoid X receptor (FXR), pregnane X receptor (PXR), and constitutive androstane receptor (CAR) [2]. The biochemical signal transduction pathway of vitamin D is fairly well-known: activated VDR is bound to the VDR response element (VDRE) site of DNA in cell nucleus and when vitamin D binds to VDR, it heterodimerizes with retinoid X receptor (RXR) and starts to modulate gene transcription. In addition to its role in gene transcription regulation, various extra-genomic actions are associated with the effect of vitamin D.

Based on human 25-hydroxy-vitamin D concentration ([25(OH)D]), the indicator of vitamin D status, optimal vitamin D supply, insufficient vitamin D level, and vitamin D deficiency can be defined [7]. Low vitamin D levels and vitamin D deficiency are associated with adverse cardiovascular risk factors, such as hypertension, myocardial infarction, peripheral arterial disease, and heart failure [8]. The potential targets of vitamin D can be the contractile activity of vascular smooth muscle cells, the cholesterol uptake by macrophages, vascular calcification, structure and function of endothelial cells, systemic factors promoting atherosclerosis, inflammatory processes, oxidative stress, and remodeling factors [9]. Insufficient vitamin D level or vitamin D deficiency can facilitate atherosclerosis through vascular inflammation, endothelial dysfunction, foam cell formation, smooth muscle cell proliferation. In case of inadequate vitamin D supply, the antihypertensive properties and the beneficial effects of calcitriol are insufficient [10]. According to a large systematic review and meta-analysis that has examined the effects of vitamin D supplementation on markers of vascular function in human trials, microvascular function showed modest improvement [11]. An angiotensin-II-induced hypertension animal model resulted in pronounced vascular biomechanical, contractility, and pharmacon reactivity changes. Sex differences in the impairments of coronary arterioles have been found [12,13]. It is also well known that cardiovascular risk shows gender difference. Women are protected from cardiovascular events during the reproductive ages because of the advantageous effects of estrogens, but men have relatively higher cardiovascular risk [14]. A 22-year follow-up study showed that higher vitamin D intake is associated with lower cardiovascular risk in men but not in women. It cannot be excluded that it is a dose dependent effect: low doses might be ineffective [15]. While the relationship between vitamin D supplementation and cardiovascular outcomes is still not finally settled, it seems to be certain that vitamin D deficiency can be considered an independent cardiovascular risk factor and can be related to higher risk of cardiovascular events [16]. The potent antioxidant effects of vitamin D have already been described: downregulation of the intracellular oxidative stress-related activities, supporting the oxidation and reduction control and upregulation of the certain antioxidants and anti-inflammatory cytokines. In contrast, a vitamin-D-deficient state causes oxidative stress through the enhanced intracellular oxidative damage and apoptosis, loss of redox control, and disturbed production of antioxidants and anti-inflammatory agents [17]. 

In order to reveal the relationship between vitamin D deficiency and increased cardiovascular risk in male gender, our aim was to investigate and describe the early biomechanical, structural, and possible oxidative–nitrative stress-related changes of small intramural coronary arterioles to vitamin D deficiency in male rat model, further revealing the relationship between increased cardiovascular risk and vitamin D deficiency. 

## 2. Materials and Methods 

### 2.1. Animals

All procedures conformed to the Guide for the Care and Use of Laboratory Animals published by the US National Institutes of Health (8th edition, 2011) and the EU-conform Hungarian Law on Animal Care (XXVIII/1998). The Institutional Animal Care and Use Committee of Semmelweis University and Hungarian authorities approved the study protocol (PEI/001/820-2/2015).

According to the results of the preliminary Power analysis that has been done during the experiment design, 11 animals were selected into each group, altogether twenty-two, 100–140 g weighted, 4-week-old male Wistar rats (Semmelweis University, Charles River, Hungary) were used in this experiment. Rats were housed 4–5 together in conventional cages at a constant light-dark (12:12 h) cycle and controlled temperature (22 ± 1 °C) and humidity (56%). They were supplied with tap water and food ad libitum for the following 8 weeks. Vitamin D supplemented animals (*n* = 11, served as “Control” group) were fed with conventional rat chow (SM Rat/mouse normal diet S8106-S011, Ssniff Spezialdiäten GmbH, Soest, Germany), which was supplemented with per os vitamin D (Vigantol, 20.000 IU/mL, Merck/Serono, Mumbai, India) to reach 300 IU/kgbw daily dose that can be considered for optimal vitamin D supply [18].

The vitamin-D-deficient animals (*n* = 11, “VDD” group) received specific vitamin-D-deficient rat chow containing less than 5 IU/kg vitamin D (EF Rat/mouse VitD-free diet E15312-24, Ssniff Spezialdiäten GmbH, Soest, Germany). Blood sample measurements at termination of study have proven a 5 times reduction in serum 25-hydroxy vitamin D levels in deficient animals [19].

### 2.2. In Vitro Pressure Microarteriography of Intramural Coronary Arterioles

During the experiment, body weight was measured every week and the gain of body weight was calculated from the initial (1st week) and final (8th week) body weights. After 8 weeks, animals were sacrificed. The right carotid artery was cannulated, systolic, and diastolic blood pressures were measured in general anesthesia (Nembutal, 45 mg/kg, i.p. Ceva-Phylaxia, Budapest, Hungary). Animals were bled, and all blood from the vascular system was washed out by heparinized Krebs–Ringer solution. By careful dissection, an intramural coronary arteriole, a terminal branch of the left anterior descending coronary artery was separated with an outer diameter at preparation of about 150–200 micrometer and length over 2 mm using a zoomed stereomicroscope (Wild M3Z, Heerbrugg, Switzerland) and microsurgical devices [20]. The further section of that artery was cut off together with surrounding ventricular tissue for histological examination.

The excised coronary arteriole segments were placed into an organ chamber (Experimetria Ltd., Budapest, Hungary) filled with normal Krebs–Ringer (nKR) solution. They were cannulated at both ends with microcannulas and extended to their in vivo lengths. The composition of the nKR solution (in mM/L) was: NaCl 119; KCl 4.7; NaH_2_PO_4_ 1.2; MgSO_4_ 1.17; NaHCO_3_ 24; CaCl_2_ 2.5; glucose 5.5, and EDTA 0.034. The chamber was placed on the stage of an inverted microscope (Leica, Wetzlar, Germany). Pressure-servo pumps (Living Systems, Burlington, VT, USA) were connected to both cannulas and the arterioles were pressurized to 50 mmHg intraluminal pressure. The segments were allowed to equilibrate for 30 min at this pressure in nKR bubbled with 5% CO_2_, 20% O_2_, and 75% N_2_ containing gas mixture, and the temperature was kept at 37 °C during the whole measurement. After this incubation, the pressure was increased to 150 mmHg, then decreased to 0 mmHg. This cycle was repeated then pressure was elevated from 0 to 150 mmHg in 10 mmHg steps in nKR solution. Finally, passive conditions were studied in calcium-free Krebs solution (resulted in full relaxation) to evaluate vessel biomechanics. Spontaneous tone was calculated from nKR and calcium-free data. The vessel chamber was changed to calcium-free Krebs solution and after a further equilibration for 20 min at 50 mmHg, pressure–diameter curves were repeated and recorded as described above. The composition of the calcium-free Krebs solution (in mM/L) was: NaCl 92; KCl 4.7; NaH_2_PO_4_ 1.18; MgCl_2_ 20; MgSO_4_ 1.17; NaHCO_3_ 24; glucose 5.5; EGTA 2, and EDTA 0.025. All compounds were purchased from Sigma-Aldrich (St. Louis, MO, USA-Budapest, Hungary). Pictures of the transilluminated segments were taken during the measurement by a digital video camera (Leica DFC 320, Leica, Wetzlar, Germany) connected to the microscope. The outer and inner diameters (D_o_ and D_i_) of the vessels were measured on the magnified pictures of the arterioles by ImageJ image analysis software (Image J 1.5, National Institute of Health, Bethesda, MD, USA). For the calibration, micrometer etalon (Wild, Heerbrugg, Switzerland) was used.

### 2.3. Biomechanical Calculations

From the measured inner and outer diameters and other parameters, the following morphological, geometrical, and biomechanical characteristics of the vessels were calculated:

- Outer radius/*R_o_* (μm):
  (1) $$R_{o}=\frac{D_{o}}{2}$$
- Inner radius/*R_i_* (μm):
  (2) $$R_{i}=\frac{D_{i}}{2}$$
- Wall thickness/*h* (μm):
  *h* = *R_o_* − *R_i_* (3)
- Wall thickness/Lumen diameter ratio:
  (4) $$h/D=\frac{h}{D_{i}}$$
  where *D_i_* is the lumen or inner diameter.
- Wall cross-sectional area/*A_w_* (μm^2^):
  (5) $$A_{w}=\left(R_{o}^{2}-R_{i}^{2}\right)*\pi$$
- Tangential stress/*σ_Tang_* (kPa):
  (6) $$\sigma_{Tang}=\frac{p*R_{i}}{h}$$
  where *p* is the intraluminal pressure.
- Incremental elastic modulus/*E_Inc_*:
  (7) $$E_{Inc}=\frac{2R_{i}^{2}R_{o}}{\left(R_{o}^{2}-R_{i}^{2}\right)}\;*\;\frac{\Delta P}{\Delta R_{o}}$$
  where Δ*P* is the change in intraluminal pressure and Δ*R_o_* is the outer radius change in response to Δ*P*.
- Distensibility/*D*:
  (8) $$D=\frac{\Delta V}{V*\Delta P}$$
  where Δ*V* is the change in lumen volume relative to the initial volume *V* in response to pressure change (Δ*P*).
- Myogenic tone (%):
  (9) $$Myogenic\;tone\;\left(\%\right)=\frac{R_{Ca-free}-R_{nKR}}{R_{Ca-free}}*100$$

### 2.4. Histology and Immunohistochemistry of Coronary Arterioles

Ventricular tissue samples with coronary arteriole segments in them were freshly fixed in 4% formaldehyde. They were embedded in paraffin, sectioned and stained for elastic fibers with resorcin –fuchsin (RF) and with hematoxylin–eosin (HE). Immunohistochemistry was performed against oxidative and nitrative stress markers, such as 4-hydroxy-2-nonenal (HNE), poly(ADP)-ribose (PAR), 3-nitrotyrosine (NT) as well as for α-smooth muscle actin (α-SMA). Polyclonal rabbit Anti-HNE (1:500, Abcam, ab46545, Cambridge, MA, USA), anti-NT (1:500, Abcam, ab42789, Cambridge, MA, USA), monoclonal mouse Anti-PAR (1:500, Tulip Biolabs., Cat. #1020, Lansdale, PA, USA), and Anti-α-SMA (1:10.000, Abcam, ab7817, Cambridge, MA, USA) antibodies were applied. Secondary labeling was achieved by using horseradish-peroxidase (HRP)-labeled horse anti-rabbit and anti-mouse IgG polymer detection kit (Vector Laboratories, MP-7401, MP-7402, Burlingame, CA, USA, 30–40 min). Brown-colored 3-3′-diamino-benzidine peroxidase HRP substrate kit (Vector Laboratories, SK-4100, Burlingame, CA, USA) was used to visualize the specific antigen-labeling and blue-colored hematoxylin QS nucleus stain (Vector Laboratories, H-3404-100, Burlingame, CA, USA) was used to counterstaining. 

The stained sections were photographed with microscope coupled video-camera (Zeiss AxioImages. A1 with Zeiss AxioCam MRc5 CCD, Carl Zeiss, Germany or Nikon Eclipse NI NI-SS, 933584 with Nikon DS-Ri2 camera and NIS Elements BR image software, Nikon Corporation, Japan). On the digitized pictures, area percentage, nucleus count, and non-calibrated optical density of different parts of the vessel wall (tunica media or whole part) were measured with ImageJ image analysis software.

### 2.5. Statistical Analysis

Statistical analysis and figures were made with the help of GraphPad Prism 7.0 (GraphPad Software, San Diego, CA, USA) software. After checking Kolmogorov–Smirnov, D’Agostino and Pearson omnibus and Shapiro–Wilk normality tests, in case of data with normal distribution, we used parametric unpaired T-test with F-test, data with non-normal distribution were analyzed with non-parametric Mann–Whitney U-test. Repeated measures variance analysis (ANOVA) with Bonferroni’s post hoc test was used in case of increasing intraluminal pressures. For all statistical analyses, *p* < 0.05 was considered statistically significant. Values with normal distribution are expressed as mean ± SEM, data with non-normal distribution are expressed as median [IQR]. Significance symbols: *: *p* < 0.05; ‡: *p* < 0.01; #: *p* < 0.001.

## 3. Results

### 3.1. Physiological Parameters

There was no significant difference in the initial and final body weight and the gain of weight of the animals at termination of study (in median [IQR]: initial body weight in grams: 125.7 [117.7–127.7] g and 120.0 [113.2–129.5] g; final body weight in grams: 425.4 [384.3–468.8] g and 411.2 [385.4–424.0]; gain of body weight in grams: 304.5 [258.1–343.1] g, and 290.5 [265.4–295.5] g; gain of body weight in percentage in mean ± SEM: 246.4 ± 11.81% and 229.4 ± 10.76% for control and VDD groups, respectively, non-significant), indicating that the growth of the adolescent–young adult rats during 8 weeks were the same, and it was not influenced by our different treatments. Arterial blood pressures were not significantly different either (systolic blood pressures (in mean ± SEM): 118 ± 6 and 123 ± 5 mmHg; diastolic blood pressures: 89 ± 6 and 94 ± 4 for the control and vitamin-D-deficient groups, respectively).

### 3.2. Geometry of Coronary Arterioles

**Figure 1:**
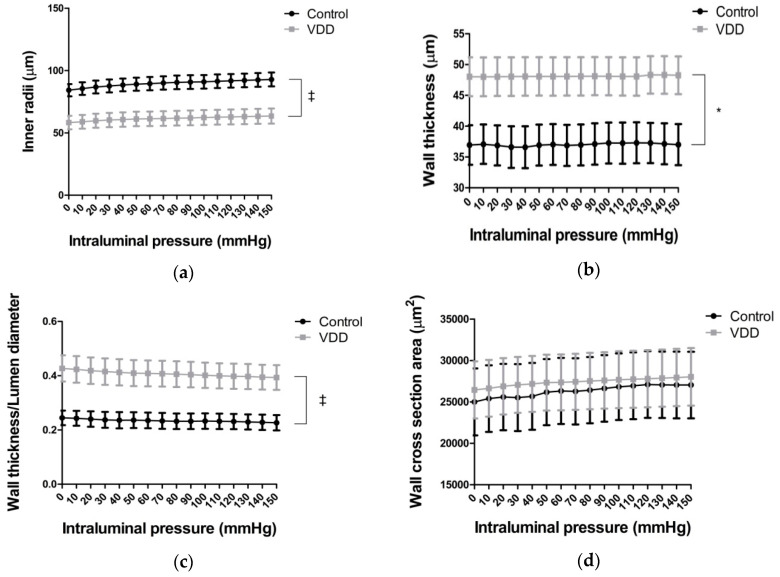
Geometry of coronary arterioles. (**a**) Inner radii (μm, *n* = 8–8); (**b**) wall thickness (μm, *n* = 8–8); (**c**) wall thickness/lumen diameter ratio (*n* = 8–8), and (**d**) wall cross-section area (μm^2^, *n* = 7–7) of coronary arterioles in calcium-free solution on 0–150 mmHg intraluminal pressures. Repeated measures ANOVA, Bonferroni. Mean ± SEM, *: *p* < 0.05; ‡: *p* < 0.01.

Significant differences (*p* < 0.01) were observed between the control and vitamin-D-deficient groups’ inner radii measured in calcium-free medium, at all intraluminal pressure levels (Figure 1a). Vitamin-D-deficient state caused significantly larger wall thickness (*p* < 0.05) and wall thickness/lumen diameter ratio (*p* < 0.01) as measured in the passive state (Figure 1b,c). However, the cross-sectional area of the wall (amount of wall material) did not change (Figure 1d).

### 3.3. Elasticity of Coronary Arterioles

**Figure 2:**
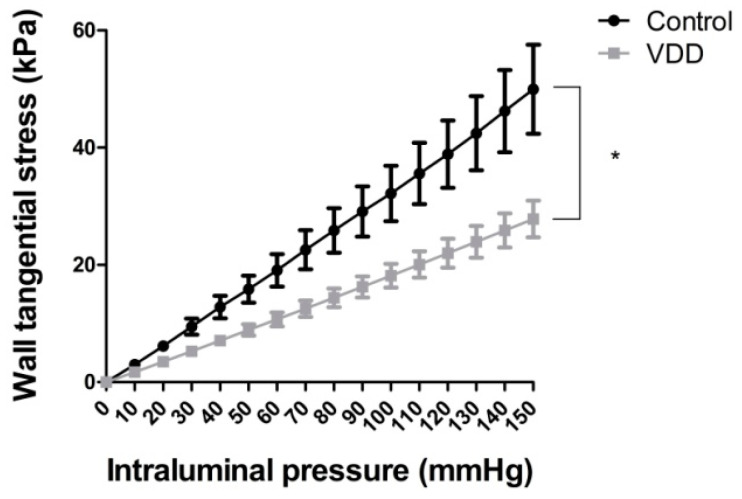
Tangential stress (kPa) of coronary arterioles in passive state repeated measures ANOVA, Bonferroni. Mean ± SEM, *n* = 8–8. * *p* < 0.05.

Tangential stress was significantly lower (*p* < 0.05) in the vitamin-D-deficient group under passive circumstances (Figure 2).

**Figure 3:**
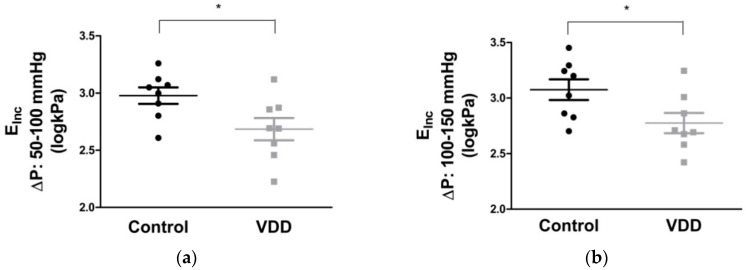
Incremental elastic modulus (logkPa) of coronary arterioles in the (**a**) 50–100 and (**b**) 100–150 mmHg intraluminal pressure ranges in passive state. Unpaired *T*-tests. Mean ± SEM, *n* = 8–8. *: *p* < 0.05.

Incremental elastic modulus, which describes the elastic stiffness of the wall material of the coronary arterioles, significantly decreased (*p* < 0.05) in vitamin-D-deficient rat arterioles when measured at higher pressures (Figure 3a,b).

Distensibilities measured in passive conditions did not change significantly (in mean ± SEM: 11.2 ± 2.1 × 10^−5^ kPa^−1^ and 10.2 ± 1.5 × 10^−5^ kPa^−1^ for control and vitamin-D-deficient groups, respectively) in the range of 50–100 mmHg.

### 3.4. Myogenic Tone of Coronary Arterioles

**Figure 4:**
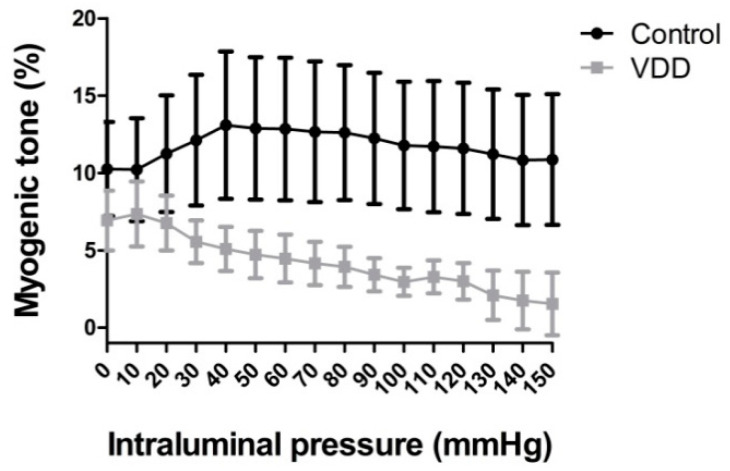
Spontaneous tone of coronary arterioles (%, *n* = 6–6). Repeated measures ANOVA, Bonferroni. Mean ± SEM.

Myogenic tone of coronary arterioles from vitamin-D-deficient animals seemed not to be able to keep the tone at high pressures; however, this difference did not reach the level of statistical significance (Figure 4). 

### 3.5. Histology and Immunohistochemistry of Coronary Arterioles

**Figure 5:**
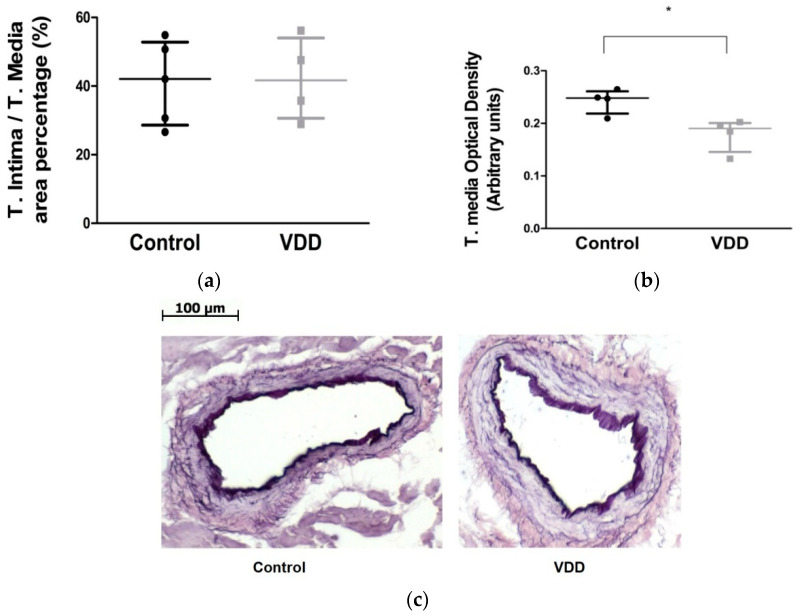
Histological properties of coronary arterioles. (**a**) T. Intima/T. Media area percentage (%, *n* = 5–4); (**b**) T. Media optical density (*n* = 4–4) of resorcin–fuchsin-stained coronary arterioles. Mann–Whitney U-tests. Median [IQR], *: *p* < 0.05; (**c**) representative photos of resorcin–fuchsin-stained male rat coronary arteriole segments from control and vitamin-D-deficient groups. Scale bar, 100 μm.

The ratio of the cross-section of the tunica intima/tunica media was not different between the two groups (Figure 5a). However, optical density of RF elastica stain in the media was significantly lower in vitamin-D-deficient vessels (Figure 5b). That indicates substantial structural changes in the wall.

Smooth muscle cell nuclei were counted in the medial layer on HE-stained sections, but they did not show any difference between the control and vitamin-D-deficient groups (nucleus count/1000 μm^2^ in median [IQR]: 6.83 [5.94–10.05] and 6.57 [5.27–8.41], respectively, *n* = 5–4, for control and VD-deficient groups, non-significant).

**Figure 6:**
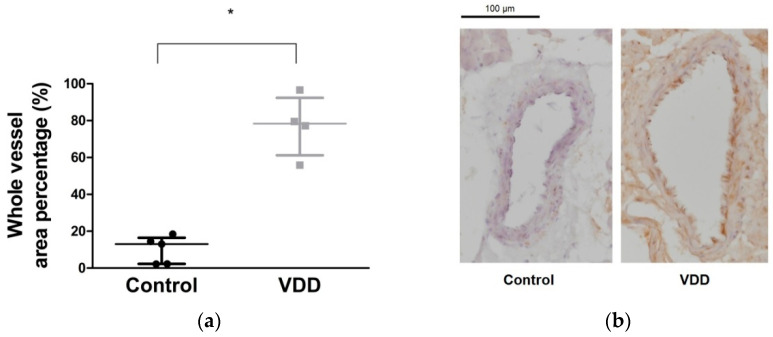
Results of anti-4-hydroxy-2-nonenal (HNE) immunohistochemical staining. (**a**) Percentage area of coronary arteriole cross-sections positively staining with anti-HNE antibodies. Mann–Whitney U-test. Median [IQR], *n* = 5–4. *: *p* < 0.05; (**b**) representative photos of HNE immunohistochemistry sections of male rat coronary arteriole segments. Scale bar, 100 μm.

The oxidative stress and lipid peroxidation marker, 4-hydroxy-2-nonenal (HNE) level was significantly elevated (*p* < 0.05) in the vitamin-D-deficient group (Figure 6a,b).

**Figure 7:**
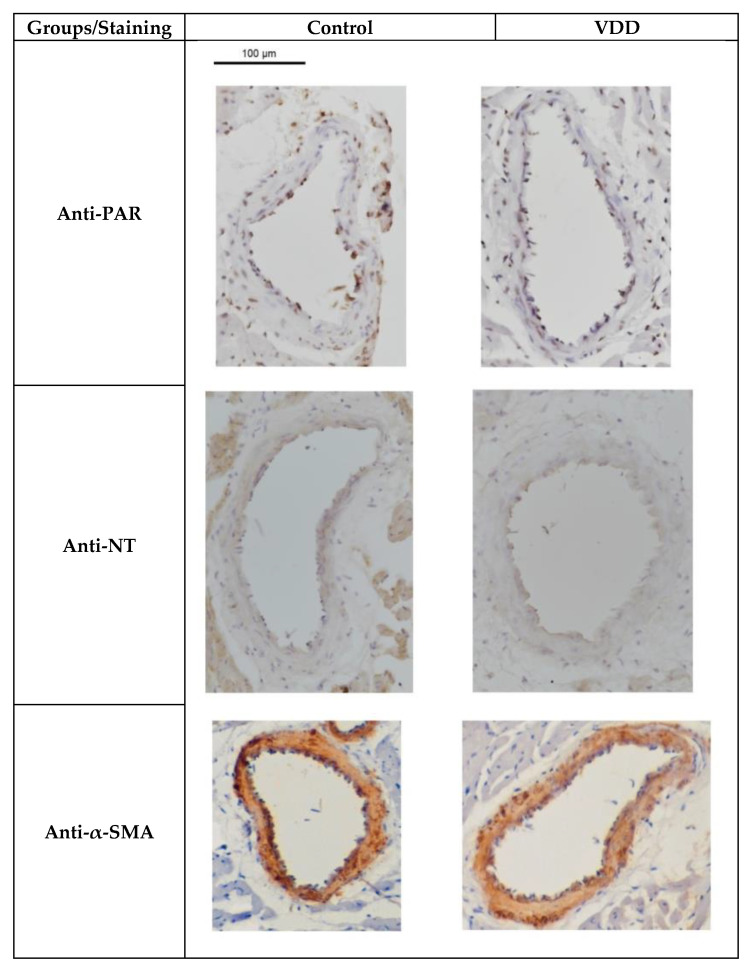
Representative photos of poly(ADP)-ribose (PAR), 3-nitrotyrosine (NT), and -smooth muscle actin (α-SMA) immunohistochemistry sections of male rat coronary arteriole segments. Scale bar, 100 μm. Abbreviation: VDD = Vitamin-D-deficient.

There were no statistically significant differences in poly(ADP)ribose, 3-nitrotyrosine, and α-smooth muscle actin immunohistochemistry staining (Figure 7).

## 4. Discussion, Limitations, and Strengths

According to our findings, an 8-week-long vitamin-D-deficient state in male Wistar rats caused significant structural, geometrical, biomechanical, elastic, and histological changes in small intramural coronary arteriole segments, although the blood pressure was unaltered. 

Lumen reduction, enlarged wall thickness, and wall thickness/lumen diameter ratio with unchanged wall cross-section area refer to inward eutrophic remodeling. Considering the structural and geometrical changes, reduced lumen diameter with increased tunica media thickness/lumen ratio are typical in essential hypertension [21]. Resistance vessels from spontaneous (genetically) hypertensive rats (SHR) have smaller lumen diameter and thicker wall and media thickness [22]. Analogous trophic vascular consequences, such as reduced lumen with increased wall thickness, were found in female rats with similar vitamin-D-deficient background [23], indicating that the pre-hypertensive adaptation in small intramural coronary arteries is independent of gender. These alterations can be considered as pre-hypertensive adaptation due to unchanged blood pressure. Based on biomechanical and elastic changes, possible background of this adaptational response that causes impairments in these vascular functions diminished tangential wall stress on higher intraluminal pressure and decreased incremental elastic modulus. 

In terms of histological changes, lowered optical density of tunica media layer with decreased elastic modulus refers to initial elastic changes in wall material and may lead to reduction in the elasticity of vessel wall in the longer term. 

Matrix metalloproteinase (MMP) family members are known as one of the major proteolytic enzymes for regulating extracellular matrix degradation and tissue remodeling. They have other non-matrix functions, such as regulation of cell surface receptors, cell–cell adhesion molecules, cytokine, clotting factors, chemokines, and other proteinases. According to their substrate-based, classification, collagenases, gelatinases, stromelysins, matrilysins membrane-type, and other type of matrix metalloproteinases can be categorized. Alternative, domain-based classification distinguishes archetypal matrix metalloproteinases, matrilysins, gelatinases, and furin-activable matrix metalloproteinases [24]. MMPs with their four types of endogenous tissue inhibitors (TIMP1-4) are responsible for extracellular matrix (ECM) homeostasis; they can modulate the amount of extracellular matrix structure components dynamically [24]. This homeostasis can be influenced by various endogenous or exogenous agents or different health states. It seems that vitamin-D-deficient state, oxidative stress, and cardiovascular diseases affect negatively this sensitive balance. Vitamin D receptor-mutant mice showed reduced elastic content in the aorta [25] and diet-induced vitamin-D-deficient mice had increased aortic expression of matrix metalloproteinase-2 and -9 compared to controls [26]. In humans, vitamin D supplementation combined with a weight-loss diet in obese vitamin-D-deficient patients significantly decreased the serum levels of MMP-9 but did not cause change in MMP-2 levels [27]. MMP-2 and MMP-9 belong to gelatinase MMP group. Gelatinases degrade not only denaturated type I collagen and native type IV collagen [28] but ficbronectin, aggrecan, and other non-ECM substrates [24]. Significantly elevated MMP-9 levels were measured in the sera of patients with coronary artery disease, and it may contribute to the pathogenesis of that illness [29]. MMPs could be used as valuable diagnostic biomarkers of stable coronary artery disease [29,30].

Nuclear factor erythroid 2-related factor 2 (Nrf2) signal transduction system is the major regulator of oxidant resistance, endogenous defense mechanisms, inflammation, and a protector of various cell types and organ systems [31]. Nrf2 regulates genes, which contain antioxidant response elements (AREs) and Nrf2 can decrease oxidative stress by regulating the expression of these genes [32]. Downregulation of this signaling pathway can lead to oxidative stress and activate MMPs, and it can work in the opposite direction: oxidative stress has an influence on Nrf2 signaling and MMP functions. Male Nrf2 knock-out (Nrf2−/−) control mice had significantly higher HNE and NT levels, moreover in these mice the level of MMP-2 and MMP-9 significantly increased in cortical brain tissue lysate compared to wild-type (WT or Nrf2+/+) control animals in traumatic brain injury animal model [31]. Oxidative stress can modulate Nrf2 signaling by causing changes in its degradation [32,33]. Increased oxidative stress can also contribute to enhanced activation of matrix metalloproteinase that may result elastinolysis and vascular remodeling [34].

Vitamin D may have a possible beneficial influence on this signal transduction pathway and its effects, however few studies investigate the link between them. In an Alzheimer’s disease rat model, vitamin D analogue treatment significantly increased the Nrf2 expression and significantly decreased a lipid peroxidation marker, the malondialdehyde (MDA) levels compared to non-treated group [35]. There is one study that hypothesizes that lithocholic acid can bind to VDR, and VDR activates SIRT1/Nrf2 signal pathway and results in beneficial effects of lithocholic acid on injured intestinal barrier function [36]. 

**Figure 8:**
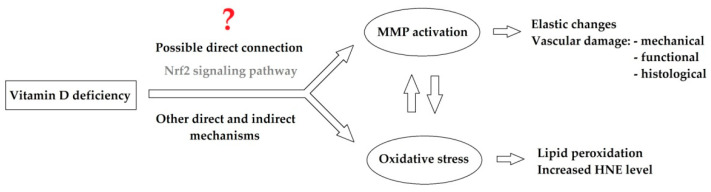
Hypothesized molecular background of the identified mechanical, functional, and histological small vessel alterations in a vitamin-D-deficient animal model. Abbreviations: Nrf2 = Nuclear factor erythroid 2-related factor 2; MMP = Matrix metalloproteinase; HNE = 4-hydroxy-2-nonenal.

According to our results, we hypothesize the role of modified activity of matrix metalloproteinases and Nrf2 molecular signaling pathway in the pathomechanism of the identified mechanical, functional, and histological vascular alterations (Figure 8). Further investigations need to identify the link between vitamin D, vitamin D deficiency, and Nrf2 signaling pathway and matrix metalloproteinase activity as well as oxidative–nitrative stress development in various organ systems, such as cardiovascular system. 

Unchanged α-SMA expression and smooth muscle cell nucleus count found in this study might indicate that the contractile part of the vessels was not affected within 8 weeks. Lowered wall stress and reduced elastic modulus of arteriole segments were shown in the coronary artery from vitamin-D-deficient female rats [23]. Cerebral artery from vitamin-D-deficient male rats had attenuated wall stress [19]. Decreasing tendency of spontaneous tone of vitamin-D-deficient group was not significant, but moving in this direction may develop into a pre-hypertensive lesion due to long-term vitamin-D-deficient state. Significantly reduced tone was observed in vitamin-D-deficient female rat cerebral arteries [37], compared to SHR rats, where coronary arterioles generated greater tone at higher pressure [38]. 

Early elastic impairments and unaltered contractile elements may explain the declining trend of myogenic tone. It is possible that in coronary resistance arteries the reduction in myogenic tone is transient due to the initial elastic changes. The structural–histological, functional, and mechanical lesions caused increased vascular resistance, and in case of stable blood pressure, the coronary perfusion diminished, which as a result, leads to the deterioration of the heart’s blood supply. Our results are consistent with Folkow’s hypotheses that not only pharmacological (renin–angiotensin system) but also various vascular mechanisms participate in the development of hypertension [39,40]. Our aim was to identify early, initial changes and the detailed background of these impairments.

Vitamin-D-independent and vitamin-D-dependent organs and tissues can be distinguished. This theory is supported by the described vitamin-D-dependent and -independent effects of the vitamin D receptor and vice versa, the VDR-dependent and independent actions of this vitamin or “pro-hormone” [41]. In accordance with these results, on the one hand, in a vitamin-D-deficient state the vitamin D receptor expression of some organs and tissues may rise, thus the vitamin D supply does not affect the target-organ function, and it becomes independent from the actual vitamin D concentrations. On the other hand, in case of vitamin-D-dependency, the organ or tissue cannot adapt by receptor-upregulation to the concentration changes, so it will be sensitive and vulnerable to vitamin D deficiency. According to our previous results from animal experiments, small vessels from the heart and brain are vitamin-D-dependent tissues, because in extreme vitamin-D-deficient milieu, they go through major changes [18,19,23,37,42], but these alterations are depending on which region the vessels are originated from. Both vitamin D deficiency and toxicity can induce smaller carotid artery diameter and larger wall thickness in male Wistar rat models within 4 weeks [43]. Otherwise, short-period intraperitoneal injection of 150.000–200.000 IU/kg [44] and 700.000 IU/kg [45] vitamin D with intragastric administration of high-lipid emulsion within approximately 3 months was used to model atherosclerosis in rats. In humans, coexistence of hypertension and vitamin D deficiency enhanced the probability of developing small vessel disease; moreover, patients with this disease were more likely to be male with higher blood pressure and lower 25(OH)D level [46]. Typical hypertensive alterations have not been developed yet during our treatment. 

However, in this early pre-hypertensive state, significant change in oxidative damage such as increase in immunohistochemical HNE levels could be detected simultaneously with the observed mechanical alterations. Oxidative–nitrative stress can cause lipid peroxidation, protein damage, DNA/RNA damage and changes in appearance of several biomarkers. HNE is a commonly used lipid peroxidation marker, and NT is a widely used protein damage marker [47]. Vitamin D has an effect both on oxidative [17] and nitrosative/nitrative stress. Low (<100 IU/kg) vitamin D dietary intake caused significant increase in the level of 3-nitrotyrosine in the male rat brain [48]. Vitamin-D-deficient female rats showed elevated NT expression in liver and ovaries [49]. Accumulation of HNE-modified macromolecules significantly influences several cell functions, interactions, and metabolism: DNA, RNA, protein synthesis inhibition, mitochondrial dysfunction, protein aggregation, cell cycle arrest, cell death, and impaired DNA-repair mechanisms have been found [50]. In the serum of patients with abdominal aortic aneurism, the protein bound HNE was significantly higher compared to control patients, which supports the pathogenic role of oxidative stress in this vascular disease [51]. In our experiments, the increased HNE level referred to incipient lipid peroxidation, because at the same time, the peroxynitrite and other reactive free-radical production were unaltered (PAR and NT did not change). In a similar animal model, a vitamin-D-deficient diet caused increased vascular oxidative stress in aortic rings of male Wistar rats [52]. It has been described that increased lipid peroxidation and HNE production correlate with various vascular illnesses, such as atherosclerosis, diabetes, and pregnancy-related disorders [53]. Based on these findings, lipid peroxidation may be the first step that may trigger and cause the early, initial damages. The possible molecular connection between vascular dysfunction and oxidative damages may include changes in the Nfr2 signaling pathway and the increased activity of matrix metalloproteinases.

An interesting hypothesis is that the efficacy of vitamin D supply and the severity of vitamin D deficiency may show gender and individual differences. Based on animal studies, it seems that in males, higher exogenous vitamin D intake may be required to reach and maintain the same, optimal target range of vitamin D than in females [18,19]. This observation suggests gender and individual differences in vitamin D synthesis, activation, or metabolism. A human cross-sectional study has found association between lower 25(OH)D levels and lower sex-hormone binding globulin and higher free testosterone levels in both women and men [54]. May the strong (testosterone, 5-dihydrotestosterone) and weak androgens (dehydroepiandrosterone, androstenedione) activate the 24-hydroxilase enzyme more than estrogens, and as a consequence, may vitamin D inactivation become faster in males? Alternatively, the reason of this variance is in the possible differences in the estrogen and androgen steroid receptor signal transduction that links to vitamin D physiology? It is known that VDR, PXR, and CAR receptors belong to same nuclear receptor sub-family [2], and estrogen and androgen receptors are also steroid nucleus receptors, and it is possible that there might be some cross-reactions between their ligands and receptors. Another attractive theory is that in the same individual the vitamin D requirement changes during variant life periods and, as a result of illnesses, different metabolic states. In males, the correlation of vitamin D state with different lifetimes and normal conditions (intrauterine life, mini-puberty, puberty, adulthood, andropause, aging male) as well as pathological alterations and various illnesses is still a less researched area that needs further thorough investigations.

Limitations and strengths of the study: our immunohistochemical oxidative damage marker measurements were semi-quantitative. Moreover, our animals were young, thus comparison with middle-aged or elderly humans is not feasible. We examined segments from hardly accessible and verifiable small intramural coronary arterioles. The low availability of coronary arteriole tissues hindrance us to perform more quantitative measures of oxidative damage markers. Further detailed investigations in animal models and human population are required to understand the molecular mechanisms underlying the pathomechanism of the found alterations in different ages and various vitamin D statuses, and whether additional vitamin D supplementation following different terms of the noxa could ameliorate the changes that have been developed during vitamin D deficient condition.

In summary, our aim was to identify initial vascular changes induced by vitamin D deficiency in male gender. According to our findings, the mechanical alterations and increased oxidative damage correspond to the pre-hypertensive state despite unaltered blood pressure and myogenic tone. The observed histological and biomechanical changes could induce increased vascular resistance and, potentially, reduced ventricular tissue blood flow. 

## 5. Conclusions

This study is the first one that has investigated the changes in biomechanical and oxidative–nitrative properties of intramural coronary resistance arteries in response to vitamin D deficiency in male rodents. As a consequence of a vitamin-D-deficient state, inward eutrophic remodeling had developed with increased vascular resistance. Increased oxidative damage may play a role in development of these changes. These mechanisms can contribute to the increased incidence of cardiovascular diseases in the vitamin-D-deficient state.
